# Artificial Intelligence to Improve Clinical Coding Practice in Scandinavia: Crossover Randomized Controlled Trial

**DOI:** 10.2196/71904

**Published:** 2025-07-03

**Authors:** Taridzo Chomutare, Therese Olsen Svenning, Miguel Ángel Tejedor Hernández, Phuong Dinh Ngo, Andrius Budrionis, Kaisa Markljung, Lill Irene Hind, Torbjørn Torsvik, Karl Øyvind Mikalsen, Aleksandar Babic, Hercules Dalianis

**Affiliations:** 1 Department of Computer Science, Faculty of Science and Technology, UiT The Arctic University of Norway, Hansine Hansens vei 54, Tromsø, N-9037, Norway, 47 47680032; 2 Department of Health Data Analytics, Norwegian Centre for E-Health Research, Tromsø, Norway; 3 Department of Clinic for Surgery, Oncology and Women Health, University Hospital of North Norway, Tromsø, Norway; 4 Department of Physics and Technology, UiT The Arctic University of Norway, Tromsø, Norway; 5 Group Research and Development, DNV (Norway), Sandvika, Norway; 6 Department of Computer and Systems Sciences, Stockholm University, Stockholm, Sweden

**Keywords:** large language models, AI, ICD-10, ICD-11, clinical coding, Easy-ICD, CAC, artificial intelligence, Computer Assisted Coding, International Statistical Classification of Diseases and Related Health Problems codes, Tenth Revision, International Statistical Classification of Diseases and Related Health Problems codes, Eleventh Revision, International Statistical Classification of Diseases

## Abstract

**Background:**

Clinical coding is critical for hospital reimbursement, quality assessment, and health care planning. In Scandinavia, however, coding is often done by junior doctors or medical secretaries, leading to high rates of coding errors. Artificial intelligence (AI) tools, particularly semiautomatic computer-assisted coding tools, have the potential to reduce the excessive burden of administrative and clinical documentation. To date, much of what we know regarding these tools comes from lab-based evaluations, which often fail to account for real-world complexity and variability in clinical text.

**Objective:**

This study aims to investigate whether an AI tool developed by by Norwegian Centre for E-health Research at the University Hospital of North Norway, Easy-ICD (International Classification of Diseases), can enhance clinical coding practices by reducing coding time and improving data quality in a realistic setting. We specifically examined whether improvements differ between long and short clinical notes, defined by word count.

**Methods:**

An AI tool, Easy-ICD, was developed to assist clinical coders and was tested for improving both accuracy and time in a 1:1 crossover randomized controlled trial conducted in Sweden and Norway. Participants were randomly assigned to 2 groups (Sequence AB or BA), and crossed over between coding longer texts (Period 1; mean 307, SD 90; words) versus shorter texts (Period 2; mean 166, SD 55; words), while using our tool versus not using our tool. This was a purely web-based trial, where participants were recruited through email. Coding time and accuracy were logged and analyzed using Mann-Whitney U tests for each of the 2 periods independently, due to differing text lengths in each period.

**Results:**

The trial had 17 participants enrolled, but only data from 15 participants (300 coded notes) were analyzed, excluding 2 incomplete records. Based on the Mann-Whitney U test, the median coding time difference for longer clinical text sequences was 123 seconds (*P*<.001, 95% CI 81-164), representing a 46% reduction in median coding time when our tool was used. For shorter clinical notes, the median time difference of 11 seconds was not significant (*P*=.25, 95% CI −34 to 8). Coding accuracy improved with Easy-ICD for both longer (62% vs 67%) and shorter clinical notes (60% vs 70%), but these differences were not statistically significant (*P*=.50and *P*=.17, respectively). User satisfaction ratings (submitted for 37% of cases) showed slightly higher approval for the tool’s suggestions on longer clinical notes.

**Conclusions:**

This study demonstrates the potential of AI to transform common tasks in clinical workflows, with ostensible positive impacts on work efficiencies for clinical coding tasks with more demanding longer text sequences. Further studies within hospital workflows are required before these presumed impacts can be more clearly understood.

## Introduction

### Background

International Statistical Classification of Diseases and Related Health Problems codes, Tenth Revision (ICD- 10) [1] plays an important role in health care. All hospitals in Scandinavia record their activity by summarizing patient encounters into ICD-10 codes. Clinical coding directly affects how health institutions function on a daily basis because they are partially reimbursed based on the codes they report. The same codes are used to measure both volume and quality of care, thereby providing an important foundation of knowledge for decision-makers at all levels in the health care service.

Clinical coding is a highly complex and challenging task that requires a deep understanding of both medical terminology and intricate clinical documentation. Coders must accurately translate detailed patient records into standardized codes, navigating the inherently complex medical language, which makes this task prone to errors and inconsistencies. Continuously evolving and progressively complex coding standards in health care, coupled with human factors, have been shown to lead to documentation of poor quality, which in turn often leads to inappropriate treatment and follow-up of patients [2,3].

Unlike in some parts of the world, like the United States and United Kingdom, where clinical coding is taken as a serious profession with an advanced training curriculum, in Scandinavia, the common practice is for junior doctors to perform the coding and for medical secretaries to quality-assure the coding. This operational workflow difference may be a partial explanation for why clinical coding practice in Scandinavian hospitals is unsatisfactory. Investigations from Norway [4] and from the other countries [5-8] have exposed similar issues. The extent of these coding issues will vary across countries and medical areas. For instance, the Swedish National Board of Health and Welfare reports that, in hospital cancer care, 22% of the main diagnoses were wrong [5], while in Norway, the Office of the Auditor General of Norway (Riksrevisjonen) reported that up to 40% of the main diagnoses for lung diseases were incorrectly coded when reviewing selected patient records from 2017 [9].

Computer-assisted coding (CAC) has been proposed as a plausible tool for solving these problems. The American Health Information Management Association defines CAC as the “use of computer software that automatically generates a set of medical codes for review, validation, and based upon provider clinical documentation” [10]. Modern CAC approaches often include AI since LLMs like ChatGPT have demonstrated impressive capabilities in natural language processing tasks. However, these generative AI models tend to perform poorly when applied to clinical coding [11,12]. This poor performance is perhaps largely explained by the vast and intricate label space of ICD codes (with thousands of specific options), and lack of localized, domain-specific, clinical data for training purposes. However, recent studies demonstrate that in some extremely limited instances characterized by narrowly defined label space or clinical problems, ChatGPT can still perform well [13].

Even though complete or fully automatic code assignment using current methods is nearly impossible, semiautomatic solutions present a practical and effective alternative. Instead of automatic assignment, these tools provide a list of code suggestions while still relying on human oversight and control over the final coding decisions. However, to date, most studies evaluating CAC systems are conducted in controlled, lab-based environments, which limits their applicability to real-world clinical settings. While these studies provide valuable insights into the potential of CAC tools, they often fail to capture the complexities of real-life settings. This gap underscores the need for more field-based research with users. This study was conducted with health staff using our CAC tool, Easy-ICD, which produces smart code suggestions based on information in the electronic health record (EHR).

### Related Work

Although research on CAC tools has a long history in Scandinavian countries [14] and elsewhere [15,16], much of the significant progress can be attributed to the recent advances in deep learning methods [17,18]. These new methods are better adept at dealing with the challenges of the large label space (tens of thousands of codes), unbalanced datasets, and long text sequences, even though they also introduce new challenges such as explainability [19]. While deep learning is a key component in recent studies, ensembles combining multiple methods are quite common [20-22]. A review by Yan et al [23] provides an overview of how methodologies evolved from purely rule-based methods to neural network-based methods. It further demonstrates how renewed interest in the problem spans different training datasets in not only major languages such as English and Chinese, but also French, Italian, Hungarian, German and Spanish [20,24]. Increased recent research activity [17,25,26] suggests that CAC systems have the potential to streamline the coding process and reduce the burden on clinical coders, especially when processing high volumes of patient records.

However, the evidence regarding the overall utility of CAC systems is not conclusive. Some studies have reported improved accuracy when a CAC system is used but without any time savings, and others have also reported time savings when a CAC system is used but without improving accuracy. For instance, Zhou et al [27] used regular expressions to implement a CAC system to enable automatic ICD-10 coding. The tool was developed and used for 16 months 2017‐2018. A total of 160,000 codes were automatically assigned and compared with manual coding. The finding was that the automatic system was 100 times faster in ICD-10 coding compared to manual coding while still maintaining good coding quality. The *F*_1_-score of the system was relatively low, around 0.61.

In contrast, another study by Chen et al [18] constructed an automatic coding tool using the Bidirectional Encoder Representations from Transformers (BERT ) algorithm trained on patient records from one Taiwanese hospital. The discharge summaries contained 14,602 labels in total. The ICD-10 auto-coding tool predicted ICD-10 codes with the best *F*_1_-score of between 0.715 and 0.618. The tool was used in a user study where the tool did not really decrease coding time but the coding quality increased significantly from a median *F*_1_-score of between 0.832 nd 0.922.

While heterogeneity in different studies partially explains the inconclusive evidence, there are some factors that are not fully explored in the literature, including the complexity of the clinical texts. It is conceivable that the variability of text length is a major factor when considering how a human versus a machine performs. In this study, we focus on text length variability in an attempt to explore the conditions under which CAC systems are most effective.

### Objective

This study seeks to provide evidence on the potential of AI to enhance clinical coding practices, thereby improving operational efficiency and data quality in healthcare. We evaluate the effectiveness of the tool, under two conditions: (i) when the clinical notes are long versus and (ii) when they are short. We denote length by the number of tokens or words in each clinical note, and do not consider other factors such as complexity of the medical case, terminology difficulty or required medical knowledge. Using word count allowed us to focus specifically on the impact of note length on coding performance, without the confounding influence of variations in medical case complexity.

Word count directly reflects the amount of text that coders need to process. Even a simple medical case can generate a lengthy note if there is a lot of descriptive text, detailed history, or extensive documentation. While word count is a simplified measure, it is a more objective and easily quantifiable measure that is effective, since longer texts generally require more time to read than shorter texts, regardless of other possible complexity factors.

We aim to answer the following research question: Does the Easy-ICD tool increase the speed and quality of ICD-10 coding compared to traditional manual coding for both long and short Swedish discharge summaries?

## Methods

### Trial Design

We followed the CONSORT- EHEALTH (Consolidated Standards of Reporting Trials of Electronic and Mobile Health Applications and Online Telehealth) template [28] for reporting our study, which is a CONSORT extension for studies reporting eHealth-related interventions (see checklist in Checklist 1).

**Figure 1.**
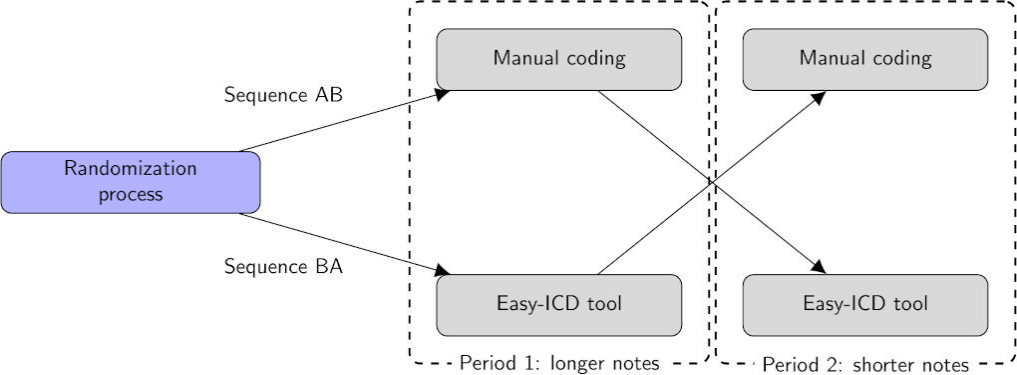
Crossover study design, where Period 1 contains 65% of the total word count and Period 2 contains 35% of the total word count. ICD: International Classification of Diseases .

The study is designed as a crossover randomized controlled trial with an allocation ratio of 1:1. The study design is illustrated in Figure 1, where participants alternate between using manual coding and the Easy-ICD tool to code long and short notes, with random assignment to different sequences to control for period and order effects. Period 1 contains 65% of the total words (mean 307, SD 90; tokens per note), and Period 2 contains only 35% (mean 166, SD 55) tokens per note), where each period has 10 notes. This fixed-order design helps assess the impact of the Easy-ICD tool on coding performance, in terms of accuracy and speed, by controlling for variation in note length and balancing the order of interventions.

Participants were randomized into two groups , Sequence AB and Sequence BA. and crossed over between Period 1 and Period 2. This ensures that each participant codes both long and short notes but experiences the interventions in a different order to balance out any learning effects or biases related to note length. There was no additional model training, bug fixes, downtime, or content changes to the intervention, and no unexpected events were noted after the trial commenced.

### Participants

#### Eligibility Criteria for Participants

Participants were required to have computer literacy and prior experience in medical coding or relevant health care documentation to ensure familiarity with the coding tasks. We did not specify a minimum requirement for prior experience. The participants were nurses, coding experts, and physicians from Sweden and Norway. The focus was on professional coders, who usually are nurses trained in coding. Individuals with any medical condition or cognitive impairment that could affect their ability to complete the coding tasks were excluded from participation.

The study was organized as a web-based intervention and participants were recruited in Norway and Sweden through email targeting health care institutions in these countries. Each participant received a web link to the study and was allowed to complete it using a device of his or her choice whenever it was convenient. There were no face-to-face components, and the study was completely anonymous in the sense that the study team did not get to know which recipients participated.

In addition to study instructions to the participants, we limited multiple submissions through cookies. The recruitment email provided information about the project and the study goals, and we also offered them 2 cinema tickets each. Since we targeted practicing professionals, we emphasized that participation was anonymous. In the email, we also provided the link to the consent form (Multimedia Appendix 1).

#### Settings and Location Where the Data Was Located

The web application, AI model, and all data were hosted at the Norwegian Centre for eHealth Research in the north Norway regional health authority. Participants were made aware of the institution conducting the study through recruitment email. The URL of the web application was also a subdomain of the institution. The primary outcomes were assessed through detailed logs of user actions in the browser.

### Interventions

**Figure 2.**
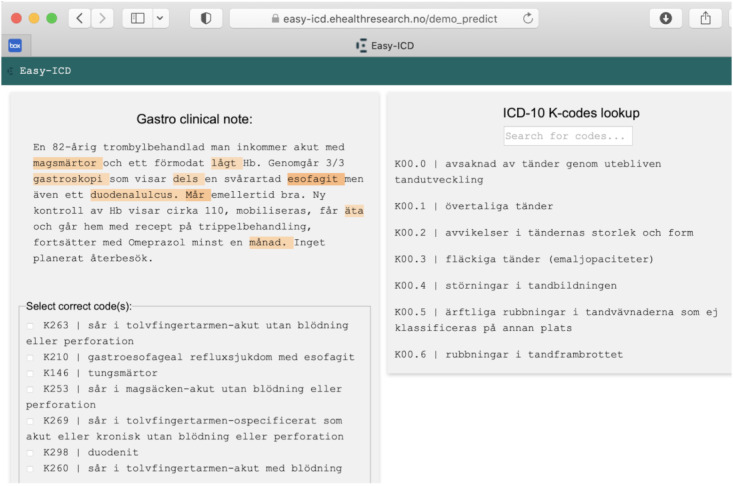
Screen dump of the Easy-ICD demonstration.

The coding tool, Easy-ICD, was developed by co-authors of this paper and ownership lies with the regional hospital, the University Hospital of North Norway. While the AI model has restricted availability, other nonsensitive components, like the web application, are open-source. This tool was developed using co-design methods involving clinical coders, clinicians, and data scientists over a period of about 3 years. The tool was designed as a web application using a typical Model-View-Controller software design pattern [29]. The model has two components; a BERT-based clinical LLM as described in [30], and a fuzzy logic component described in [21]. The view is composed of custom HTML5 templates, while the controller that connects the user interface is based on Flask, a Python (Python Software Foundation) framework, see Figure 2, for a screen dump of the demonstration (Multimedia Appendix 2). We chose a simple web interface design so that participants could focus on the task with minimal distractions. All development ceased at the protocol stage [31] and the version of the tool was “frozen” and no additional revisions or updating was done.

The dataset used for training of the LLM model behind Easy-ICD-tool is the Stockholm EPR Gastro ICD Pseudo Corpus II fine-tuned on the SweDeClin-BERT Swedish clinical language model (415 unique K-codes, 113,175 patients and 81,089 discharge summaries with a total of 56 million tokens) [30]. We used the full dataset with 415 codes for training the model. However, in terms of label imbalance, approximately 80% of the data only uses approximately 20% of the 415 codes, therefore most of the codes were underrepresented. In terms of the technical validation, for the top 5 predictions, performance reached an *F_1_*-score of 0.88 for the full corpus, and 0.94 for the top 80%. Further details on this technical validation are discussed in [30]. Since training was done with real-world data, we recognize that unverified coding may introduce some uncertainty in how our model performs, especially for rare codes with limited training data.

A subset of 20 randomly selected discharge summaries from the test dataset were recoded by a senior Swedish coder for the purposes of quality-assuring the notes used in this study, that is, creating the gold standard. We acknowledge that reliance on a single coder could introduce bias, and that creating a gold standard is an inherently difficult task in clinical coding. However, for difficult cases, the Swedish coder also had a chance to discuss with a Norwegian coder.

To use our solution, participants needed to follow the Internet link with direct access to the study, but they would initially face a consent form. If they agreed to proceed, they would watch a short instructional video before the study begins (see instruction video in Multimedia Appendix 3). To use our tool in this study or as an integrated tool in an electronic health record (EHR) system, participants would not need special training, but participants needed a good understanding of the coding process. This usually means they must have had some training in coding. For a given chunk of text, our solution suggests 5‐7 codes from which the participants have to select one or more codes. We instructed them to consider only K-codes, since the notes were from the gastrointestinal department and to consider other codes if no K-code was appropriate. The number of codes that the solution suggests (5‐7 codes) was based on our discussion with clinical coders during the requirements gathering phases of the project. With 2 components to our tool (LLM and fuzzy logic), 5 suggestions come from the LLM, the other 2 from fuzzy logic, and sometimes the 2 components intersect. We figured this 5:2 ratio as optimal on our data, based on experimental testing and trial-and-error.

During the study, participants were not allowed to be disrupted; we instructed them to carry out the task in one sitting. If they were disrupted, they were to quit the study immediately. To help participants adhere to the requirements of the study, information redundancy was used; first in the consent form and also in a short video just before the coding task began. No other human assistance was available to them. However, because of the slow recruitment, we sent reminders to everyone on the mailing list because we could not tell who had already completed the study and who had not.

#### Data Collection and Management

All participant interactions with the user interfaces were logged to ensure comprehensive data collection. Every action performed by the participants, such as clicks and selections were logged. To track the duration of tasks and response times, the exact times at which each action was performed were also logged. This also includes all AI-generated suggestions and whether they were accepted, modified, or ignored by the participants. This logging was conducted in real-time and stored securely on the server, ensuring that no data were lost and that all data were immediately available for analysis.

Data management was handled with strict adherence to privacy and security protocols to protect participant confidentiality and data integrity. All communication had end-to-end encryption using transport layer security [32], a commonly used standard for securing internet communications using web browsers. To keep the data anonymous, personal identifiers were not collected. No IP addresses were collected and cookies were only used to track progress in the experiment. This ensured that individual participants could not be directly identified from the dataset. Without IP tracking or some user management software, we cannot rule out the possibility that a participant could have completed the study more than once. However, we assessed this risk as low since the participants were practicing professionals and we communicated the importance of completing the study once in one sitting.

Even though the raw data is anonymous, access to the data was restricted to authorized personnel only. A secure login system was used to manage access rights. Regular data integrity checks were performed to ensure that the data was complete, consistent, and free from corruption. Regular backups of the data were taken to prevent data loss. These backups were stored in separate, secure locations and could be restored in case of data corruption or accidental deletion.

### Outcomes

There were two primary outcome measures in this study; the time used in assigning codes, and the accuracy of the coding, all assessed during one session of 40‐60 minutes duration. Time was logged as the participants navigated through each note in the study by clicking the “Next” button. In terms of accuracy, we measured how the participants performed compared to the gold standard. One important change to the trial outcome was that we originally planned to measure usability of our solution as a secondary measure. We then considered that by the time participants reached the usability survey, they would likely be exhausted, and that might have had an impact on the result. Since we had some usability feedback during the co-design process with clinical coders, we decided to conduct a separate usability study at a later date. However, we used star-rating of usefulness as an indirect qualitative gauge of user satisfaction with the system.

### Sample Size

To calculate the sample size for the 2-sided tests used in our study, particularly for the Mann-Whitney *U* test (a nonparametric test), we needed to consider several factors, such as the desired power of the study (80%), medium effect size (0.5), significance level or the probability of a Type I error (0.05). Considering prior studies related to CAC systems, such as [27], which reported a substantial reduction in coding time, our use of a medium effect size was a conservative estimate, and only used to guide recruitment targets. The significance level (α) is the probability of a Type I error; rejecting the null hypothesis when it is true, while the power (1 - β) is the probability of correctly rejecting the null hypothesis when it is false (avoiding a Type II error). For the effect size, we used Cohen *d* for Mann-Whitney *U* test, which quantifies the difference between groups. Given that smaller effect sizes require larger samples, we used medium effect size.

For a rough sample size estimate using Cohen effect size (d), the formula for the sample size per group (n) in a nonparametric context can be approximated using the following formula:


$$n=\left(Z\alpha /2+Z\beta \right)/d^{2}$$

Where:

- Zα/2 is the Z-score for the desired significance level (for *α*=.05, Z=1.96),
- Zβ is the Z-score for the desired power (for 80%, Z=0.84),
- *d* is the estimated effect size (Cohen *d*).

Z-values measure the necessary spread (SDs) on a standard normal curve to meet our specified significance level and statistical power, and together Zα/2 and Zβ establish the threshold of certainty required to detect a true effect of side d. Given that we assume a medium effect size d=0.5, power of 80%, and α of .05, approximately 32 observations per group would be needed in each of Period 1 and Period 2. Recruitment strategies included contacting hospitals and health authorities, announcing the study at coding seminars and conferences, and using popular science media, social media, and other electronic platforms for advertising and outreach.

### Randomization

The randomization sequence was generated using alternate switching between 0 and 1 to ensure randomness and minimize allocation bias, and participants were assigned to Group 1 (sequence AB) and Group 2 (sequence BA) in a 1:1 ratio. The randomization process was implemented as follows: after obtaining informed consent, the participant was randomly assigned the next number in the 0‐1 toggle sequence to reveal the participant’s group assignment. The group assignment was then recorded in the study log. Participants were not informed of their group allocation, but they were made aware that they would be allocated to either group.

### Allocation Concealment Mechanism

Since the previous allocation was logged, the next user would be allocated the next in sequence, but the participants would not know to which group they would be allocated until the study begins.

### Implementation

The random allocation sequence was generated automatically by the web application. Intervention or group assignment was done automatically. A research assistant helped recruit participants through email.

### Blinding

Blinding was not used in this study because the intervention involved a clearly distinguishable user interface, making it impossible to mask from the participants and investigators. The nature of our solution required participant awareness for proper adherence. In addition, the primary outcomes were objective measurements not subject to observer bias, further reducing the need for blinding. Thus, while blinding is a valuable technique to minimize bias, its application was neither feasible nor necessary for the integrity and validity of our particular study.

### Similarity of Interventions

Participants interacted with 2 different user interfaces that were designed to be visually and functionally similar to ensure a consistent user experience across both intervention and control groups. Both interfaces featured the same layout, design elements, and core functionalities to minimize any potential confounding variables related to usability or user satisfaction.

The primary distinction between the two interfaces was the inclusion of an artificial intelligence (AI) feature in the intervention interface. This AI feature provided real-time suggestions and recommendations to the users based on the clinical text they are reviewing. Conversely, the control interface did not include this AI suggestion feature; users reviewed the clinical texts and assigned codes without receiving any automated suggestions.

Maintaining similar user interfaces ensured that any differences observed between the two groups could be attributed to the presence or absence of the AI feature, rather than other extraneous factors related to interface design or functionality.

### Statistical Methods

In this study, we opted not to perform commonly used statistical analyses in crossover studies, which typically account for the dependency between periods. This lack of paired or crossover analysis also means that the primary benefits of a conventional crossover framework are not realized and can therefore be viewed as a compromise that sacrifices some statistical efficiency.

The primary reason for this decision is because we needed to consider the impact of clinical note length, and we assigned longer notes to Period 1 and shorter notes to Period 2. Therefore, we treated the 2 periods as independent since the levels of difficulty of the clinical notes varied significantly between the two periods. It would have been inappropriate to assume that performance in one period could be directly compared to performance in the other, as this could introduce bias related to task difficulty rather than the intervention itself.

Treating the periods independently enabled us to evaluate the effect of each intervention (Manual coding vs Easy-ICD) under different task conditions (long vs short notes) without conflating these factors. If we assume independence between periods, we avoid the complexities introduced by possible carryover effects and ensure a clearer, unbiased assessment of each intervention within each period.

We used the Mann-Whitney *U* test to analyze coding time for each period separately. The Mann-Whitney *U* test was appropriate because our data had a non-normal distribution. Also, the test is robust to outliers, which allowed us to compare the performance between the two groups without assuming normality. This approach provided a more straightforward interpretation of the results, given the distinct nature of the periods. We excluded incomplete data from our analysis, and since the study included a single sitting, we also did not consider attrition. Statistical analyses were performed using statistical software R and StatsDirect (version 4.0.4). A CI if 95% and *P* value of less than .05 was considered statistically significant.

### Ethical Considerations

We sought ethics approvals from Norway for the study and from Sweden for the use of Swedish AI models and data. This user study is part of the ClinCode Project approved by The Regional Committee for Medical and Health Research Ethics, Norway (260972). This research has been approved by the Regional Ethical Review Board in Stockholm Dnr 2007/1625-31/5 with the amendment by the Swedish Ethical Review Authority Dnr 2022-02386-02, and under permission number 2019‐05679, with the amendment no 2022-02389-02.

The first page on the intervention website was a consent form, based on a template from the Norwegian Centre for Research Data, which provided information about (1) purpose of the project; (2) institution responsible for the project; (3) why the user is being asked to participate; (4) what the user is expected to do as a participant; (5) participation as voluntary; (6) user personal privacy, when and what happens with their data; (7) information about user rights (under the General Data Protection Regulation); (8) who to contact for more information or concerns; and (9) the final section where the user clicks on “Consent” or “Decline.”

In addition to this information on the consent page, we also provided a short instructional video after consent was recorded as accepted. The study was completely anonymous, and cookies were only used to track progress during the study.

Participants were offerred two cinema tickets each through the recruitment email.

## Results

### Participant Flow

This study used a 1:1 crossover design involving 17 participants from an initial invitation pool of over 100 coders. Each participant interacted with both the intervention and control interfaces in 2 separate periods, ensuring that all participants experienced both conditions. The order of exposure to the interfaces was randomized to control for potential order effects.

Participants were randomly assigned to one of two sequences; the first sequence (A|B) using the control interface, followed by the intervention interface, and the second sequence (B|A) first using the intervention interface, followed by the control interface. Participants in each sequence switched over between Period 1 and Period 2.

**Figure 3.**
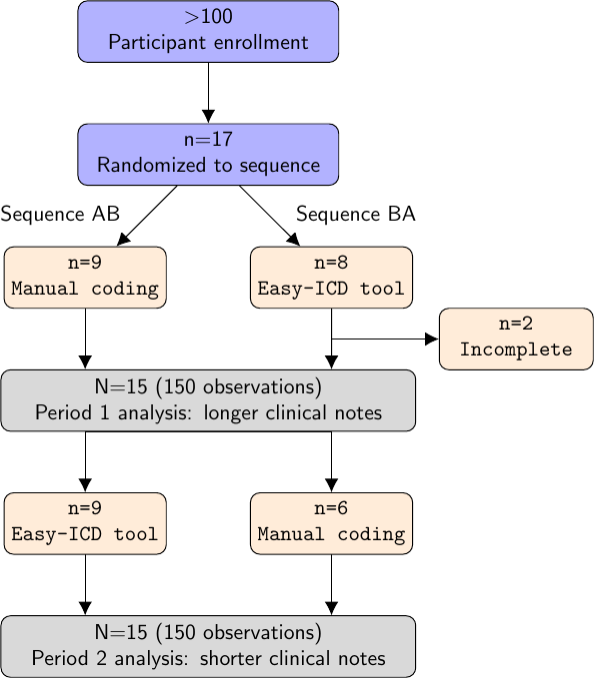
Crossover study design illustrating the AB|BA sequences, where Period 1 contains 10 long clinical notes and period 2 has an additional 10 short clinical notes, for a total of 300 observations in this study.

As illustrated in the flow diagram in Figure 3, of the 17 participants who enrolled, 15 completed both periods of the crossover study with valid data (data available in Multimedia Appendix 4). The data from these 15 participants were included in the final analysis. Since each participants coded 20 clinical notes, there was a total of 300 observations or coded texts.

### Losses and Exclusions

During the data collection, one participant’s data were deemed invalid due to incomplete data logging, and the other participant only generated a random sequence but went no further.

### Recruitment

Participants of the user study were recruited through acquaintances from our professional network in Sweden and the diagnosis-related group Forum, a network of professionals dedicated to enhancing the practice of diagnosis-related group classification in Norway. The participants were given a link to the user study along with the instructions.

Recruitment commenced in November 2023 and continued on a rolling basis until May 2024. We had to stop the recruitment because the project funds had run out and it was difficult recruiting more participants. This is because our primary target audience is practicing professional coders, and our speculation was that uncertainty regarding privacy may have resulted in a relatively low response rate. We also have some anecdotal evidence of coders expressing concerns that new AI systems may take over their jobs, and this may have caused some anxiety among potential participants.

### Baseline Data

Table 1 shows the baseline demographic and characteristics of the participants. There were 7 Norwegian and 8 Swedish coders. The majority (n=11) had more than 5 years coding experience, while only one had less than one year’s experience.

**Table 1.** Per group characteristics of the 15 participants.

Description	Participants
High experience (>5 y)	
Group 1	6
Group 2	5
Low experience (<5 y)	
Group 1	3
Group 2	1
Norwegian participants	
Group 1	6
Group 2	1
Swedish participants	
Group 1	3
Group 2	5

### Numbers Analyzed

The total number of participants was 17, but 2 had incomplete data. The analysis of primary outcomes therefore included 15 participants, with Group 1 (A|B sequence) consisting of 9 participants and Group 2 (B|A sequence) consisting of 6 participants (see Figure 3). For the secondary outcome (user satisfaction), only 55 out of a possible 150 clinical notes were rated.

### Outcomes and Estimation

#### Median Coding Time

**Figure 4.**
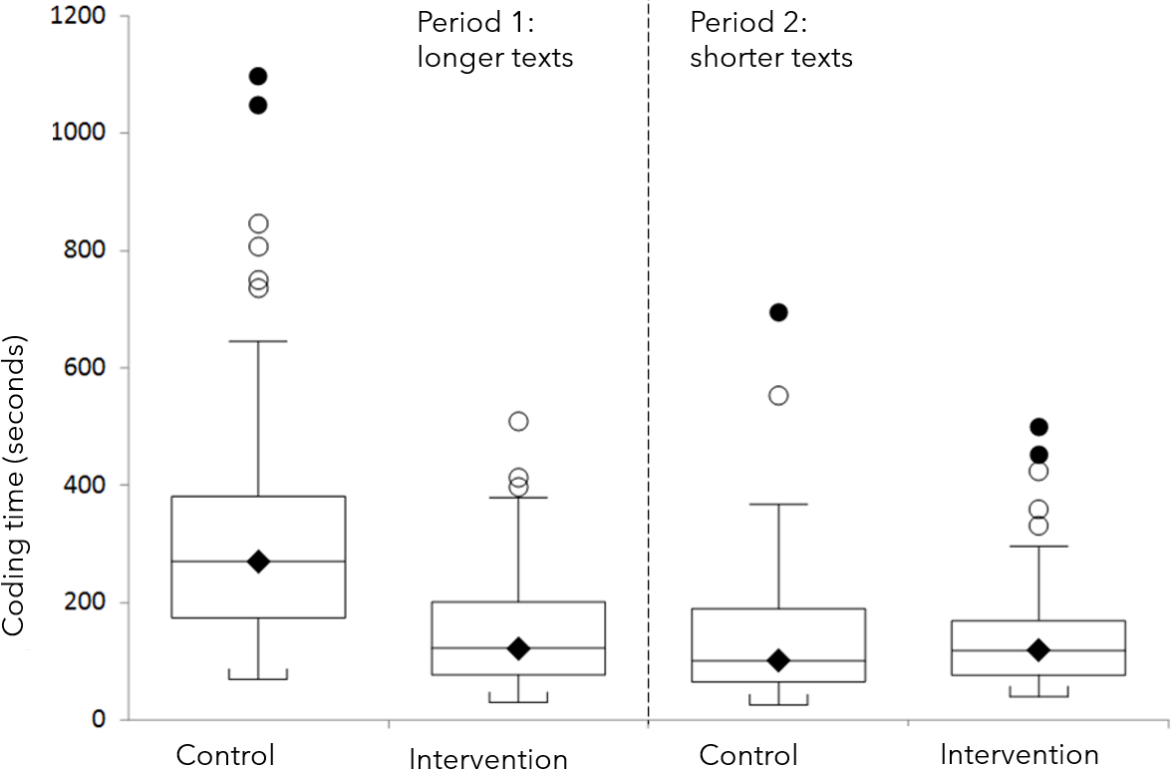
Clinical coding time (sec) for long and short clinical texts. The boxplot shows outliers (hollowed circle) and extreme outliers (solid circles).

In terms of the descriptive statistics, the control had a mean coding time of 242.6 (192.3 SD) seconds while the intervention had 147 (94.5 SD) seconds . Due to the presence of outliers and the nonparametric nature of the data distribution, we used the Mann-Whitney U test to analyze the data. The Mann-Whitney U test is a nonparametric test that does not require the assumption of normality and is less sensitive to outliers and imbalanced sample sizes. Data distribution and outliers are illustrated in the box plot in Figure 4. The test is specifically designed to assess whether the median difference between the observations is significantly different from zero. This allowed us to effectively compare the performance metrics between the two interventions for each participant.

As shown in Table 2, for the longer clinical texts in Period 1 (1-10), the median difference in coding time duration between using our solution and not using our solution was 123 seconds at 95% CI (81-164) and a 2-sided *P*<.001. This represents an approximate 46% reduction in median coding time when our solution was used. For the shorter clinical texts in Period 2 (11-20), the median difference was 11 seconds at 95% CI (−34 to 8) and a 2-sided *P*<.25, indicating an insignificant difference.

**Table 2.** Mann Whitney *U* test for code assignment time duration.

Attribute	Value
Period 1: control	
Sample size, n (%)	90 (60)
Median (IQR)	270 (174-381)
Period 1: intervention	
Sample size, n (%)	60 (40)
Median (IQR)	121.5 (76.5-201)
Period 1 results	
Total sample size	150
2-sided *P*	*<.*001
Median difference (95% CI)	123 (81-164)
Period 2: control	
Sample size, n (%)	60 (40)
Median (IQR)	101.5 (65-189.5)
Period 2: intervention	
Sample size, n (%)	90 (60)
Median (IQR)	119 (76-169)
Period 2 results	
Sample size (total)	150
2-sided *P*	.25
Median difference (95% CI)	−11 (−34 to 8)

#### Clinical Coding Accuracy

Testing coding accuracy outcomes for significance based on 2 independent proportions, we discovered that the improvement in performance was not statistically significant, as shown in Table 3.

**Table 3.** Two independent proportions for coding accuracy.

Attribute		Value
Period 1: control		
Sample size, n (%)		90 (60)
Accuracy, n (%)		56 (62.22)
Period 1: intervention		
Sample size, n (%)		60 (40)
Accuracy, n (%)		40 (66.67)
Period 1 results		
Sample size (total)		150
2-sided *P*		<.50
Accuracy difference (95% CI)		−0.044 (−0.195 - 0.114)
Period 2: control		
Sample size (%)		60 (40)
Accuracy (%)		36 (60)
Period 2: intervention		
Sample size (%)		90 (60)
Accuracy (%)		63 (70)
Period 2 results		
Sample size (total)		150
2-sided *P*		17
Accuracy difference (95% CI)		−0.1 (−0.255 to 0.054)

#### User Satisfaction

In terms of the user satisfaction with the provided suggestions, participants were slightly more satisfied with the suggestions during the longer notes (4 out of 5 star rating, n=23) compared to suggestions for shorter notes (3.7 out of 5 star rating, n=32). However, it should be noted that it is only about a third (55 out of a possible 150, 36.7%) of the clinical notes whose suggestions were rated. Therefore, the results should be interpreted with caution since there is a risk of response bias.

No ancillary analyses were performed.

#### Harms

No harm was recorded. Due to thorough testing and simplicity of the design, we did not encounter any technical problems or privacy breaches. However, some indications from staff involved in the project pointed out the problem of the study being perceived as some kind of indicator of how participants performed on their normal jobs, hence the slow recruitment.

## Discussion

### Overview

Results emerging from our study show that the Easy-ICD tool was more useful for longer clinical notes compared to shorter clinical notes. These results are not surprising, since we initially hypothesized that such an AI assistive tool could be most useful for longer clinical notes and that the varying reports in the literature could possibly be partially explained by this factor.

Our study confirms that tools such as our Easy-ICD have the potential to contribute to decision-making and to reduce the excessive burden of documentation on health care staff. Through automating the discharge summary coding process, such tools are expected to improve the speed and quality of the coding, thus facilitating more efficient health care delivery. This automation not only saves time, but it also ensures consistency in coding practices, leading to improved patient care and streamlined administrative processes.

### Interpretation/Principal Findings

We set out to answer whether our Easy-ICD tool could help reduce coding time and increase the quality of ICD-10 coding compared to traditional manual coding for both long and short Swedish discharge summaries. The reduction in median coding time was only statistically significant for long notes, suggesting that such assistive tools are comparatively more appropriate for complex tasks. We also noted that accuracy improved in both long and short notes, albeit not significantly, when our tool was used. This suggests that even though participants were quick to pick out codes for short texts, those that used our tool had better accuracy even for shorter texts, and this is something that needs further investigation with a larger sample size and a larger code space beyond K-codes.

Two possible explanations for the nonsignificant results on accuracy are that the code space was comparatively smaller (K-codes), thus also less challenging, and that the participants were highly motivated individuals with a high level of skill, and some of them were possibly in leadership positions responsible for coding.

Study findings may have been influenced by factors such as coder experience; a small sample size meant we could not analyze the influence of experience on the results, and this could be an opportunity for further study.

### Limitations

Perhaps the most obvious limitation is the small sample size. We compensated for this with multiple observations per participant, but this also introduces a possible risk of Type I errors. We did not apply any correction for multiple testing in our analysis, but future studies with larger sample sizes should consider adjusting *P* values using methods such as Bonferroni correction to account for multiple testing.

Also, since tasks were completed sequentially, cognitive carryover effects such as learning or fatigue may have influenced performance. Future studies should consider using washout periods to minimize cognitive carryover.

Another limitation of the trial could be selection bias, as participants probably had much higher confidence and affinity for testing new technology, and they were recruited from a specific pool of health care settings in Norway and Sweden.

There is also a risk of order effect or learning effect bias in a crossover design where the sequence in which participants receive the intervention may influence their response or performance. For example, participants may become more proficient and improve their coding skills due to repeated exposure to the task or the introduction of the intervention. This could lead to better outcomes in the later period of the trial.

Another limitation is that the Easy-ICD tool only predicts chapter XI codes, the so-called K-codes. This is a very limited scope of the overall ICD-10 coding system. Perhaps including other chapters would have presented more realistic and possibly also more challenging scenarios for coders. We leave this question as a possible object of future inquiry.

### Generalizability

In a routine application setting, our tool would be integrated into an EHR system; not much else would change in terms of functionality, except for the EHR’s user interface. The successful application of Easy-ICD to improve the efficiency of clinical coding in the study suggests that similar AI tools could be beneficial in health care systems in general. The findings of this study highlight the potential of AI interventions to improve clinical coding practices, regardless of geographical location, opening the door for generalizability to other Scandinavian countries that use similar workflows in their health care systems.

Successfully demonstrating the potential of assistive tools in clinical coding may also present opportunities for porting our tools to support ICD-11. Even though world governments are gearing towards implementing this new version, anecdotal evidence suggests that health care staff are less accepting of this new version. Assistive tools, such as the one presented in this study, may be the key to lowering key adoption barriers associated with the transition to ICD-11.

However, we do acknowledge that the limitations we discussed have to be addressed, and more studies with bigger sample sizes are required before we can fully understand generalizability in this context.

### Conclusions

Our results highlight the potential of assistive tools, particularly for coding longer notes, which may require greater effort and time. This has important practical implications for the use of AI in clinical coding, as these findings demonstrate that assistive technology can be effective productivity tools for reducing the excessive burden of administrative documentation. This is particularly relevant in health care where manpower is limited and accurate task completion is critical. Overall, the study demonstrates the value of AI in augmenting human performance, providing a compelling case for the broader adoption of AI-assisted interfaces to enhance productivity in clinical coding. Globally, our results demonstrate that such AI tools have the potential to reduce the effort or cognitive load related to the coding task, and thus reduce one of the major barriers associated with the adoption of more complex and detailed classification systems like the new ICD-11.

## Supplementary material

10.2196/71904 Multimedia Appendix 1 The consent form given to participants just before they could proceed to the study.

10.2196/71904 Multimedia Appendix 2 Some screenshots from the Easy-ICD tool used in this study.

10.2196/71904 Multimedia Appendix 3 Instruction video shown after consent and right before the study commences (Audio track removed for copyright reasons).

10.2196/71904 Multimedia Appendix 4 Raw data from the study.

10.2196/71904 Checklist 1 Consort-ehealth V1.6.

## Abbreviations

AI: artificial intelligence
CAC: computer assisted coding
EHR: electronic health record
EPR: Electronic patient record
ICD- 11: International Statistical Classification of Diseases and Related Health Problems codes, eleventh revision
ICD-10: International Statistical Classification of Diseases and Related Health Problems codes, tenth revision
LLM: Large language model
