# Supplementary material for: Artificial Intelligence to Improve Clinical Coding Practice in Scandinavia: Crossover Randomized Controlled Trial
Source: J Med Internet Res. 2025 Jul 3;27:e71904. doi: 10.2196/71904 (PMC12244276; doi:10.2196/71904)
Supplement: Multimedia Appendix 1 [file jmir-v27-e71904-s001.pdf]

Are you interested in taking part in the research project

”ClinCode: Computer-Assisted Clinical Coding for improving efficiency and quality in healthcare”?

This is an inquiry about participation in a research project where the main purpose is to use AI to improve the quality of ICD-10 coding. In this letter we will give you information about the purpose of the project and what your participation will involve.

This research is approved by the Swedish Ethical Review Agency Dnr 2022-02386-02, and the Regional Comittee for Medical Research Ethics Northern Norway application number 260972.

Purpose of the project

This project has two broad objectives. First, this project aims to increase clinical coding quality for Norwegian/Swedish clinical text by matching current best systems for English.

Secondly, the project aims to help coders reduce the time required to assign ICD-10 codes.

Who is responsible for the research project?

Norwegian Centre for E-health Research is the institution responsible for the project. More information about the ClinCode project can be found here.

Why are you being asked to participate?

You received this invitation because you agreed to be part of the study. The selection criteria include someone working with (or who has worked with) ICD-10 coding as a clinical coder or healthcare staff in Norway or Sweden. The study aims to recruit at least 30 participants.

What does participation involve for you?

After you consent to participate, you will go through **20 Swedish** clinical texts. You will be randomly assigned a group, either Group 1 or 2. The difference in the two groups is that one group first uses our computer assisted clinical coding (CAC) system, Easy-ICD, for half of the clinical text, before switching to coding without our CAC system for the rest of the clinical texts. The other group first codes without the CAC system for the first half of the clinical texts, and then code using our CAC system for the remaining half.

The study is estimated to last 40-60 minutes.

Participation is voluntary

Participation in the project is voluntary. If you chose to participate, you can withdraw your consent at any time without giving a reason. There will be no negative consequences for you if you chose not to participate or quit during the experiment. All information about you is anonymous, and will therefore not affect your work situation.

Your personal privacy – how we will store and use your personal data

We will only use your personal data for the purpose(s) specified in this information letter. We will process your personal data confidentially and in accordance with data protection legislation (the General Data Protection Regulation and Personal Data Act).

- The experiment is completely anonymous. All data collected and published scientific articles do not tie back to you as a participant.
- Researchers in the project will have access to the anonymized experiment data to perform analyses

What will happen to your personal data at the end of the research project?

The project is scheduled to end in March 2024. Since the collected data is entirely anonymous, the data may be kept or deleted, at the discretion of the project manager.

Your rights according to law:

So long as you can be identified in the collected data, you have the right to:

- access the personal data that is being processed about you
- request that your personal data is deleted
- request that incorrect personal data about you is corrected/rectified

- receive a copy of your personal data (data portability), and
- send a complaint to the Data Protection Officer or The Norwegian Data Protection Authority regarding the processing of your personal data

What gives us the right to process your personal data?

We will not collect identifying or personal data during this experiment. Only the session_id and clinical_note_id cookies are stored in the browser to keep track of the experiment. These cookies are deleted when you close the browser. **No identifying data is collected**, therefore we have no way to connect your answers back to you.

Where can I find out more?

If you have questions about the project, or want to exercise your rights, contact:

- Norwegian Centre for E-health Research via Professor Hercules Dalianis, hercules.dalianis@ehealthresearch.no.
- Our Data Protection Officer: Øystein Hansen, oystein.hansen@ehealthresearch.no
- Data Protection Services, by email: (personverntjenester@sikt.no) or by telephone: +47 53 21 15 00.

Yours sincerely,

Professor Hercules Dalianis

(Project Manager)

Consent form

I have received and understood information about the project ”ClinCode: Computer-Assisted Clinical Coding for improving efficiency and quality in healthcare” and have been given the opportunity to ask questions.

NB 0: please note that **ALL** the 20 clinical notes will be in **Swedish**.

NB 1: **Only K-codes** are predicted, but you may propose codes from other ICD-10 chapters if you cannot find a suitable K-code.

NB 2: this study is **completely anonymous**; no identifying data about you or your institution is tracked or collected.

Approximate clinical coding experience:

- ○ Less than 1 year
- ○ 1 – 5 years
- ○ More than 5 years

Your usual language(s) for coding is:

- ☐ Swedish
- ☐ Norwegian

Accept
