# Supplementary material for: Artificial Intelligence to Improve Clinical Coding Practice in Scandinavia: Crossover Randomized Controlled Trial
Source: J Med Internet Res. 2025 Jul 3;27:e71904. doi: 10.2196/71904 (PMC12244276; doi:10.2196/71904)
Supplement: Multimedia Appendix 2 [file jmir-v27-e71904-s002.pdf]

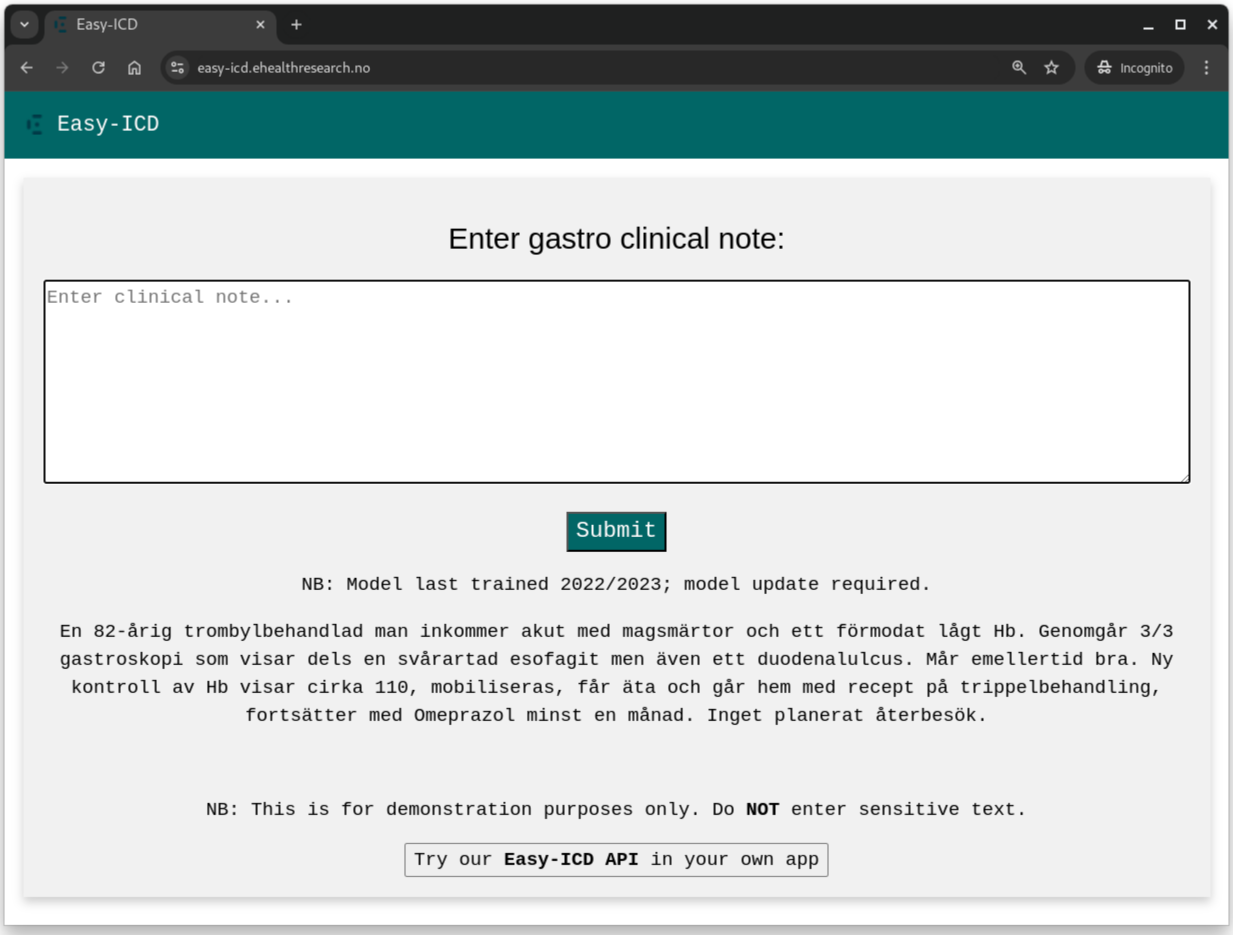

Figure 1: DEMO page as it exists today.

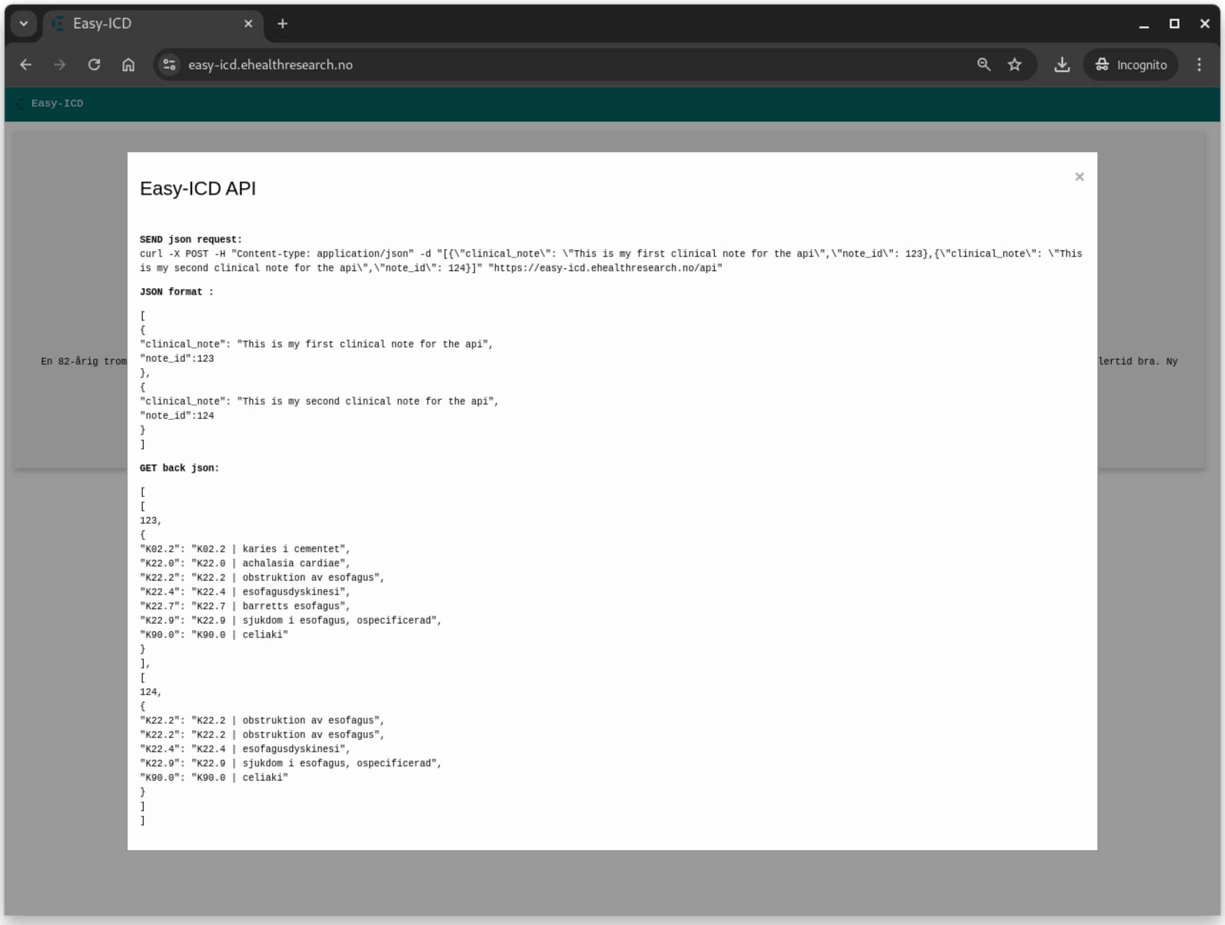

Figure 2: an API is available for the DEMO

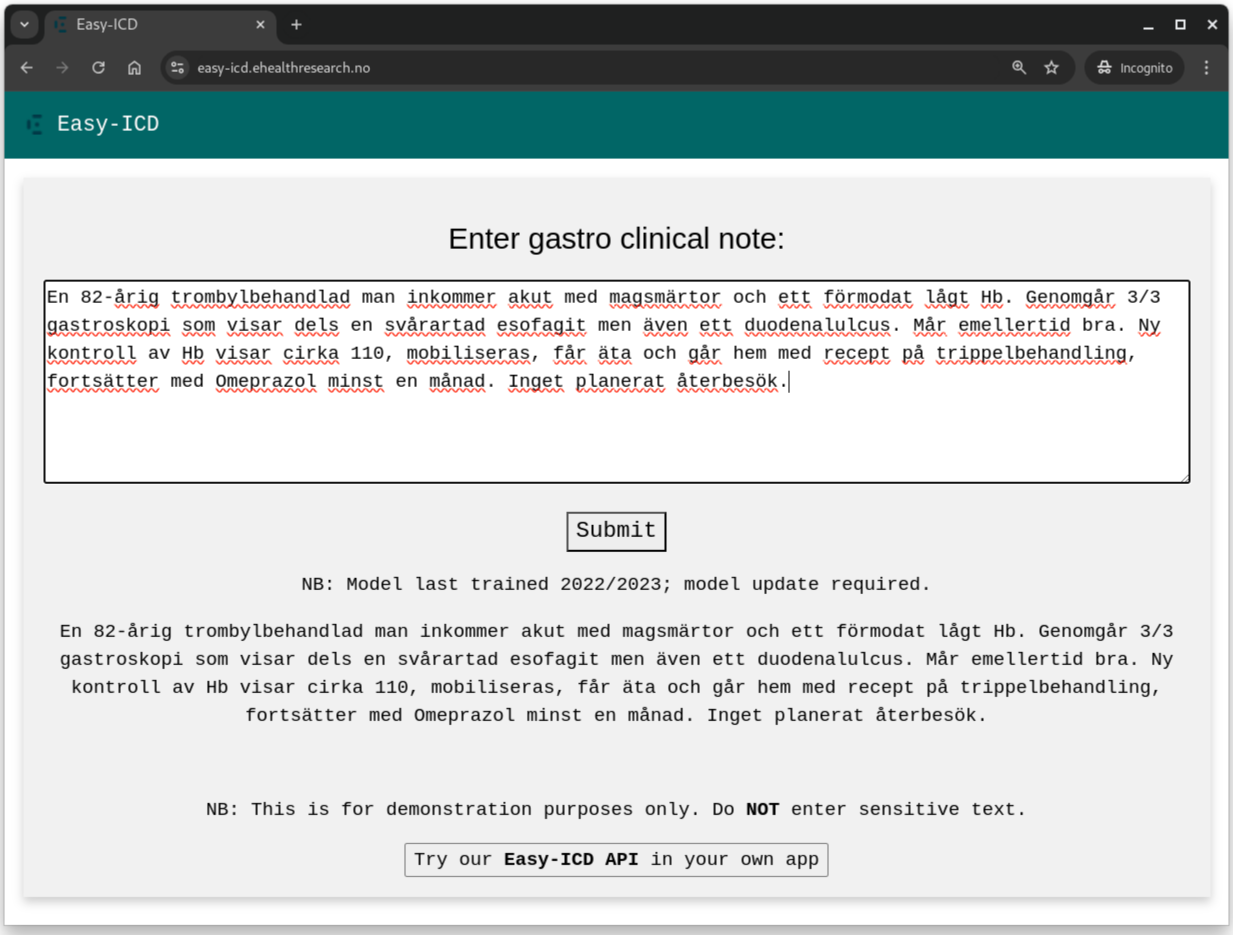

Figure 3: DEMO has sample text to use for testing purposes.

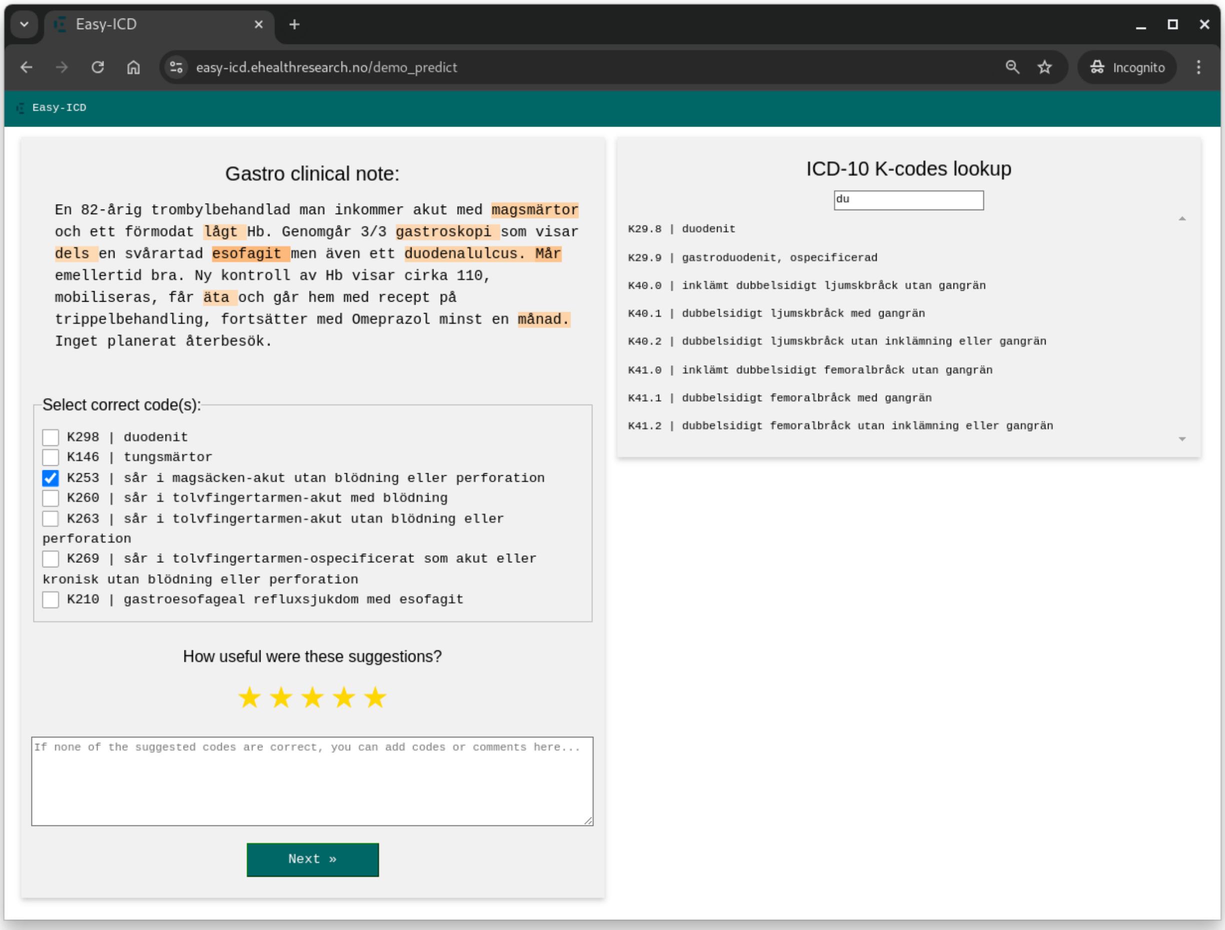

Figure 4: DEMO sample output

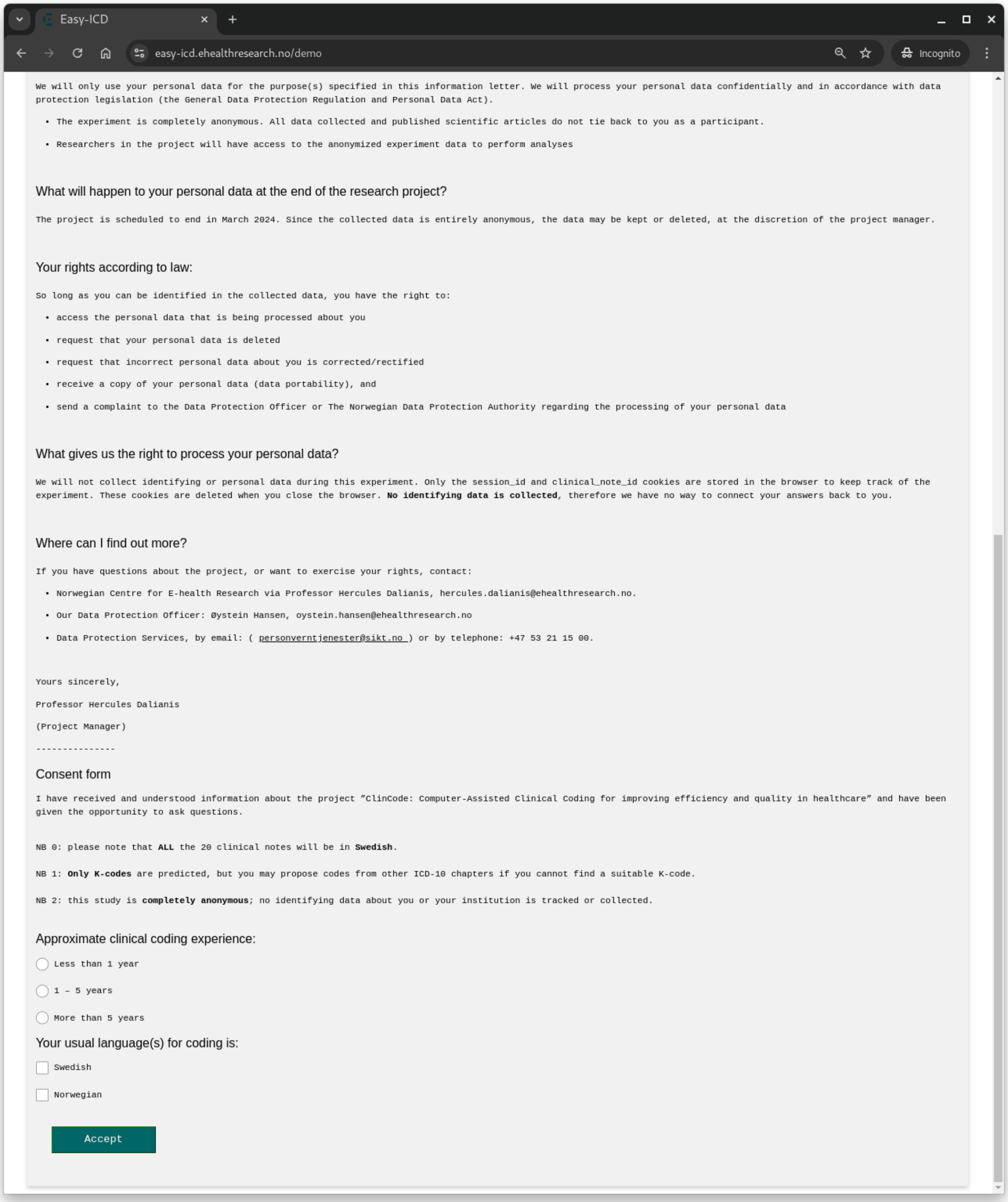

We will only use your personal data for the purpose(s) specified in this information letter. We will process your personal data confidentially and in accordance with data protection legislation (the General Data Protection Regulation and Personal Data Act).

- The experiment is completely anonymous. All data collected and published scientific articles do not tie back to you as a participant.
- Researchers in the project will have access to the anonymized experiment data to perform analyses

What will happen to your personal data at the end of the research project?

The project is scheduled to end in March 2024. Since the collected data is entirely anonymous, the data may be kept or deleted, at the discretion of the project manager.

Your rights according to law:

So long as you can be identified in the collected data, you have the right to:

- access the personal data that is being processed about you
- request that your personal data is deleted
- request that incorrect personal data about you is corrected/rectified
- receive a copy of your personal data (data portability), and
- send a complaint to the Data Protection Officer or The Norwegian Data Protection Authority regarding the processing of your personal data

What gives us the right to process your personal data?

We will not collect identifying or personal data during this experiment. Only the session_id and clinical_note_id cookies are stored in the browser to keep track of the experiment. These cookies are deleted when you close the browser. **No identifying data is collected**, therefore we have no way to connect your answers back to you.

Where can I find out more?

If you have questions about the project, or want to exercise your rights, contact:

- Norwegian Centre for E-health Research via Professor Hercules Dalianis, hercules.dalianis@ehealthresearch.no.
- Our Data Protection Officer: Øystein Hansen, oystein.hansen@ehealthresearch.no
- Data Protection Services, by email: (personverntjenester@sikt.no) or by telephone: +47 53 21 15 00.

Yours sincerely,

Professor Hercules Dalianis

(Project Manager)

Consent form

I have received and understood information about the project "ClinCode: Computer-Assisted Clinical Coding for improving efficiency and quality in healthcare" and have been given the opportunity to ask questions.

NB 0: please note that **ALL** the 20 clinical notes will be in **Swedish**.

NB 1: **Only K-codes** are predicted, but you may propose codes from other ICD-10 chapters if you cannot find a suitable K-code.

NB 2: this study is **completely anonymous**; no identifying data about you or your institution is tracked or collected.

Approximate clinical coding experience:

- ○ Less than 1 year
- ○ 1 - 5 years
- ○ More than 5 years

Your usual language(s) for coding is:

- ☐ Swedish
- ☐ Norwegian

Accept

Figure 5: Consent form is the first page to which participants were directed.

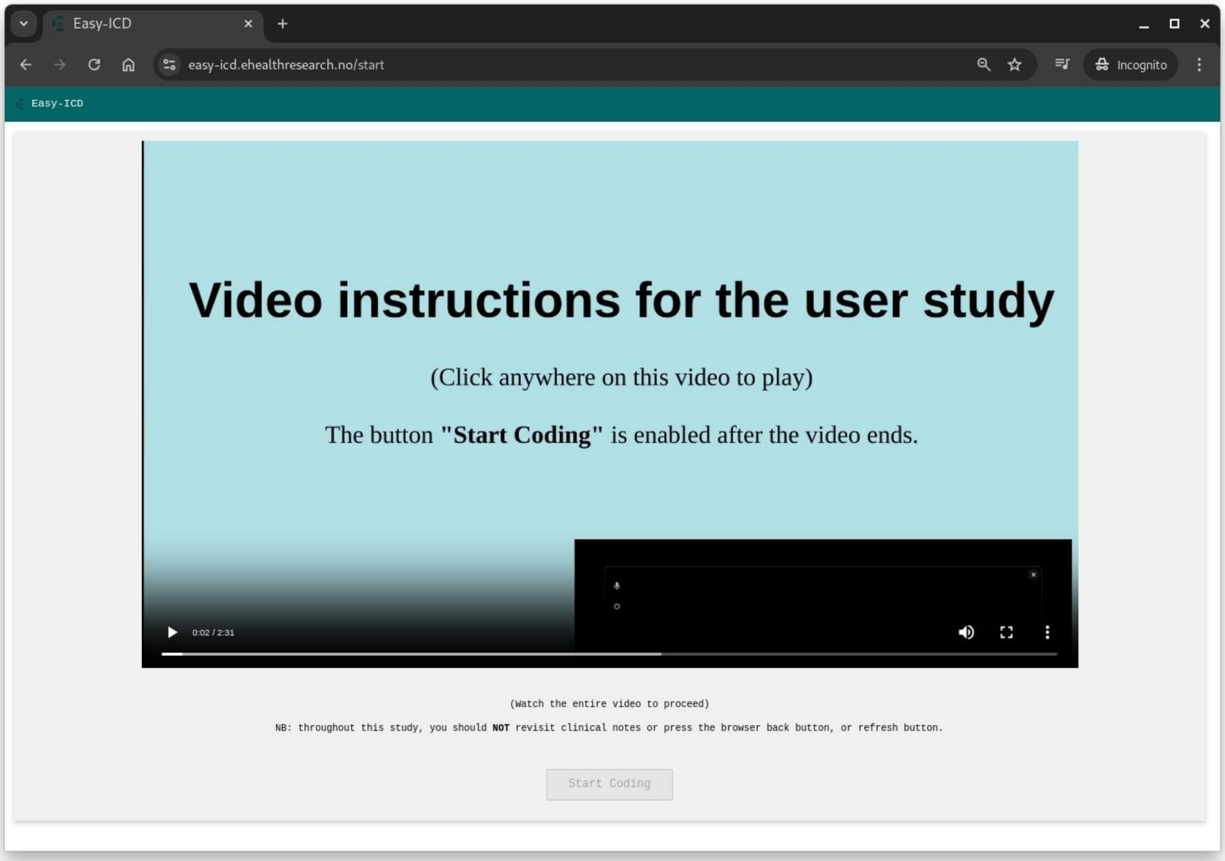

Figure 6: After consenting, the next page was the video instruction. We removed the audio track for copyright issues, but the video has subtitles in English.

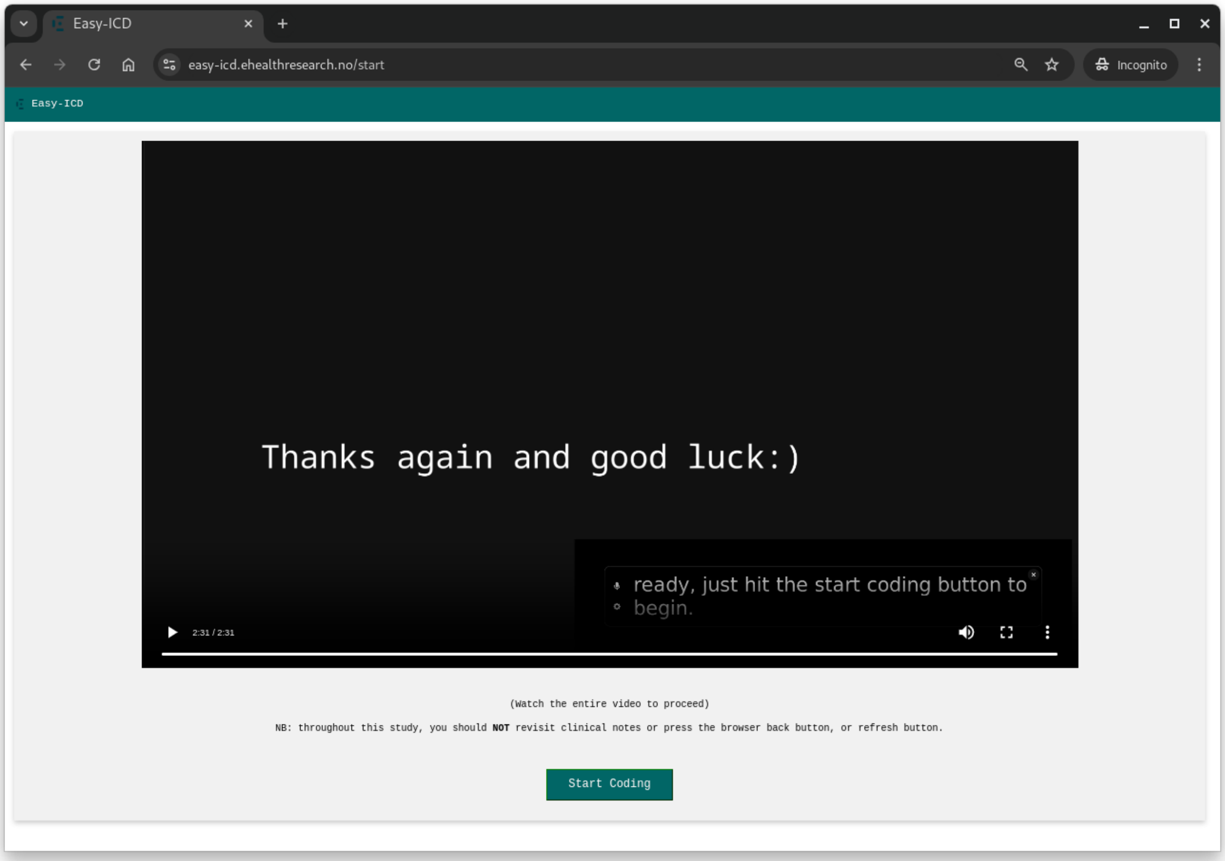

Figure 7: It is only after the participants watched the video that the “start coding” button was enabled, as a signal to commence the study.
